# Evaluation of selection program by assessing the genetic diversity and inbreeding effects on Nellore sheep growth through pedigree analysis

**DOI:** 10.5713/ajas.18.0553

**Published:** 2019-02-14

**Authors:** Satish Kumar Illa, Gangaraju Gollamoori, Sapna Nath

**Affiliations:** 1 Network Project on Sheep Improvement, Livestock Research Station, Palamaner, Sri Venkateswara Veterinary University, Tirupati, Andhra Pradesh 517408, India; 2 Indian Council of Agricultural Research, National Dairy Research Institute, Division of Animal Production, Southern Research Station, Bangalore, Karnataka 560030, India

**Keywords:** Founders, Inbreeding Coefficient, Nellore Sheep, Pedigree Analysis

## Abstract

**Objective:**

The main objectives of the present study were to assess the genetic diversity, population structure and to appraise the efficiency of ongoing selective breeding program in the closed nucleus herd of Nellore sheep through pedigree analysis.

**Methods:**

Information utilized in the study was collected from the pedigree records of Livestock Research Station, Palamaner during the period from 1989 to 2016. Genealogical parameters like generation interval, pedigree completeness, inbreeding level, average relatedness among the animals and genetic conservation index were estimated based on gene origin probabilities. Lambs born during 2012 and 2016 were considered as reference population. Two animal models either with the use of *F_i_* or Δ*F_i_* as linear co-variables were evaluated to know the effects of inbreeding on the growth traits of Nellore sheep.

**Results:**

Average generation interval and realized effective population size for the reference cohort were estimated as 3.38±0.10 and 91.56±1.58, respectively and the average inbreeding coefficient for reference population was 3.32%. Similarly, the effective number of founders, ancestors and founder genome equivalent of the reference population were observed as 47, 37, and 22.48, respectively. Fifty per cent of the genetic variability was explained by 14 influential ancestors in the reference cohort. The ratio f_e_/f_a_ obtained in the study was 1.21, which is an indicator of bottlenecks in the population. The number of equivalent generations obtained in the study was 4.23 and this estimate suggested the fair depth of the pedigree.

**Conclusion:**

Study suggested that the population had decent levels of genetic diversity and a non-significant influence of inbreeding coefficient on growth traits of Nellore lambs. However, small portion of genetic diversity was lost due to a disproportionate contribution of founders and bottlenecks. Hence, breeding strategies which improve the genetic gain, widens the selection process and with optimum levels of inbreeding are recommended for the herd.

## INTRODUCTION

Genetic diversity of a population is represented as a collection of alleles and genotypes, which generates observed differences among the individuals and populations in terms of phenotype, physiology and behavior [1] and it tends to alter under constant selection pressure and this can be monitored through the pedigree knowledge [2–4]. Up keeping the genetic diversity among the breeding individuals in closed and small population is very important as there will be an erosion of allelic distinctiveness and heterozygosity in an accelerated manner. Genetic selection and drift in the small populations leads to detrimental consequences like decreased vigor or production among animals with the increased homozygosity and loss of allelic diversity [5]. Many approaches and methods are in use to determine the extent of genetic diversity in populations [6,7].

India is bestowed with affluent genetic diversity of livestock especially sheep and ranked second in the population with 65.07 million heads [8] and possess 42 recognized sheep breeds [9] in the world. But most of the sheep genetic resources are under the process of documentation, where they exhibit better adaptation to the distinct habitat in specific agro-climatic zones of India. Nellore sheep are the tallest among the Indian breeds and prominently distributed in the semi-arid parts of southern India precisely in Andhra Pradesh state. It is choicest one for sheep production by the local shepherds, small and marginal farmers as it shown better adaptability with meager grazing resources, withstand prolonged migration and better disease resistance. Under the Network project of sheep improvement, Nellore sheep are being conserved and improved at Livestock Research Station, Palamaner, Andhra Pradesh for 25 years. This breeding center supply superior breeding rams to the local shepherds to improve their flocks.

The main objective of this study was to determine the genetic diversity and to assess the population structure of the nucleus flock of Nellore sheep based on pedigree information and to know the probable genetic losses if any and to study the effects of inbreeding on the body weights of Nellore lambs.

## MATERIALS AND METHODS

### Data collection

Data for a period of 27 years (year 1989 to 2016) were collected and utilisedfor the pedigree analysis from the breeding flock of Nellore sheep maintained at the Livestock Research Station; Palamaner, Andhra Pradesh, India (13°20′ E latitude and 78°75′ N longitude and altitude 683 m mean sea level. Reference population (1,322) was a subgroup of the main population, regarded as the cohort born during 2012 and 2016 for which various population demographic parameters were estimated (Table 1).

Four hundred females were maintained in the flock during the period and reared under semi-intensive system of management. Males were selected based on six months body weight (6MW) and their progeny performance was also considered for selection. Ten to fifteen sires were kept for breeding per year and maintained 1:25 male to female ratio for breeding. Sires used for breeding were retained in the flock for at least two years; the intensity of selection for males was approximately 10%. Major and minor breeding seasons were March to May and July to September, respectively during the study period. Twinning rate is very low in the flock. No selection criterion was applied for ewes. Females were bred either at an age of 15 months or after attaining 25 kg live weight. Ewes with poor growth and health were culled twice in a year.

Lambs were fed concentrate supplements *ad libitum* from 10 days after birth till weaning at an age of 3 months. After weaning, lambs were fed with *ad libitum* green fodder, dry hays of horse gram and alfalfa and 300 g/d/head concentrate supplement. After attaining 6 months of age, sheep were kept under grazing for 8 hours, but grazing time varied with season and ambient temperature. Grazing area consisted with deciduous vegetation and fodder trees like Subabul (*Leucaena leucocephala*), Neem (*Azadirachta indica*) and Avisa (*Sesbania grandiflora*). Flock was supplemented with 300 g/d/head concentrate mixture in the evening hours. Apart from grazing, fodder tree loppings and hays of (Stylo grass) *Stylo hamata*, (Cow pea) *Vigna unguiculata*, (Horse gram) *Macrotyloma uniflorum* and (alfalfa) *Medicago sativa* were also fed to animals.

### Statistical methods

ENDOG version 4.8 was employed for the analysis of pedigree and estimation of parameters based on gene origin probabilities [10]. The depth and wholeness of pedigree was determined by estimating the equivalent number of generations and it was estimated by tracing back the each ancestor in the pedigree history with numerous generations back.

The average relatedness (AR) coefficient of any individual is explained as the probability that an allele selected at random from the total population in the pedigree belongs to a particular animal [10] and it was estimated accordingly. Hence, the AR coefficient is equated as an account of the animal in the whole pedigree disregard of its pedigree information.

In reference population, effective number of founders and ancestors is useful in assessing the genetic history. The effective number of founders is characterized as the number of equally contributing founders that would be expected to produce the same genetic diversity as in the population under study [11]. This is computed as:

$${\text{f}}_{\text{e}}=\frac{1}{\sum_{k=1}^{f}{\text{q}}_{\text{k}}^{2}}$$

Where, q_k_ is the probability of gene origin for ancestor k [9] suggested the effective number of ancestors (f_a_) which reflects the minimum number of animals (founders or non-founders) required to estimate the genetic diversity of the population under study and it is a useful measure to know the bottlenecks in the population which are the primary reason for the genetic erosions in captive and domestic populations. It is estimated as:

$${\text{f}}_{\text{a}}=\frac{1}{\sum_{j=1}^{a}{\text{q}}_{\text{j}}^{2}}$$

Where, q_i_ is the marginal contribution of ancestor j, which demonstrates the genetic contribution of an ancestor that is not explained by earlier ancestor. In general, the effective number of ancestors should be smaller than the effective number of founders due to bottlenecks that reduce the genetic variability.

*F* is defined as the probability that an individual has two identical alleles by descent, and is computed following [10]. The change in inbreeding (Δ*F*) is estimated for each generation using the formula as suggested by Meuwissen and Luo [11] and revised by Lacy [12].

$$\Delta F_{i}=1-\sqrt[{\text{t}-1}]{1-{\text{F}}_{\text{i}}}$$

Where, F_i_ is the individual inbreeding coefficient and t is the equivalent complete generations for this individual. The estimate of effective population size (N̄_e_) [13], called realized effective size by González-Recio et al [13], was computed from $\bar{\Delta F_{\iota }}$ by averaging Δ*F_i_* of the n individuals included in a given reference subpopulation, as N̄_e_ = 1/2Δ*F̄*. The standard error was obtained as: $\sigma {\bar{\text{N}}}_{\text{e}}=\frac{2}{\surd n}{\bar{\text{N}}}_{e}^{2}\sigma \Delta F_{i}$ with n is the reference population size and σΔ*F_i_* the standard deviation of Δ*F_i_*.

Founder genome equivalent (*f*g) is defined as the number of founders that would be expected to produce the same genetic diversity as in the population under study if the founders were equally represented and no loss of alleles occurred [14]. It is obtained by the inverse of twice the average co-ancestry of the individuals included in a pre-defined reference population [15].

Genetic conservation index (GCI) [6] proposed a method for estimation of GCI, the calculation utilizes genetic contributions of all founders of the population.

$$\text{GCI}=\frac{1}{\Sigma \;pi2}$$

Where, pi is the proportions of genes of founders in the pedigree of an animal. The GCI index is particularly useful in conservation programs, in which the prime objective is to retain all the alleles from the base population. Hence, the best animal is the one that possess higher GCI value which indicates that the animal has received the contributions from all the ancestors and founders equally.

These parameters were estimated for each individual: i) the number of fully outlined generations, detailed as the number of generations delineating the offspring of the furthest generation where the ancestors of second-generation individuals are known. Ancestors with unknown parents are considered as founders (generation 0); ii) the maximum number of generations observed; determined as the number of generations separating the individual from its ultimate ancestor. iii) Equivalent complete generations are detailed as the sum over all known ancestors of the terms calculated as the aggregate of (1/2)^n^, where n is the number of generations separating the individual from each known ancestor [16–18]. The average generation interval (GI) was pointed out as the average age of the parents at the birth of their selected offspring. The estimate of GI for all the pathways was estimated for the cohort born from 2012 to 2016, as this subpopulation is the most recent one that could engross at least a generation in the flock.

To assess the impact of inbreeding, two animal models either with either the use of *F_i_* or Δ*F_i_* as linear covariable were evaluated. Fixed effects in the model were lamb sex, year of lambing (15 levels) and parity of dam with ewe weight at lambing as a covariate. Season of lambing is ignored in the present analysis as lambing during minor season was low in number. Coefficient or change of inbreeding (*F_i_* or Δ*F_i_*) was also fitted as a linear co-variable [13]. General linear model is used in the statistical analyses to know the significance of various fixed factors including inbreeding (*F_i_* or Δ*F_i_*) on the growth traits of Nellore lambs [19]. Growth traits in the present study were birth weight (BWT), weaning weight or weight at three month (WW) and weight at six month (6MW).

## RESULTS AND DISCUSSION

**Figure 1:**
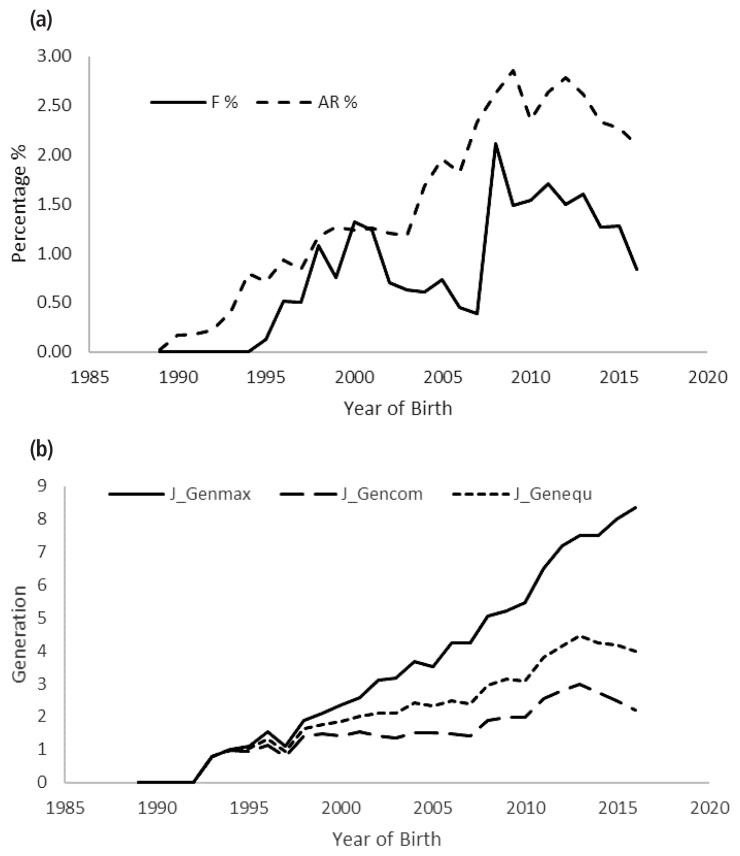
(a) Inbreeding coefficient (F %), was the probability that an individual has two identical alleles by descent. Average relatedness coefficient (AR %) was the probability that an allele selected at random from the total population in the pedigree belongs to a particular animal, year of birth for whole pedigree. The estimates were varying over the years without any consistency. (b) Average maximum generations (J_Genmax) were estimated by separating the offspring of the furthest generation, where the 2^g^ ancestors of the individual are known. Complete generations (J_Gencom) were the number of generations separating the individual from its furthest ancestor. Equivalent generations (J_Genequ) were computed as the sum over all known ancestors and these parameters were traced by year for whole pedigree. Estimates thus obtained suggested a good depth in the pedigree in terms of completeness.

In the present study, results obtained from pedigree analysis are shown in Table 1. The proportion of animals with known pedigree (with both parents) and had both the parents is 88.83%, whereas 11.17% of the lambs had unknown parents and the results suggested a good depth in the pedigree in terms of completeness. For the whole pedigree, the completeness for the first three generations was 88.93%, 64.78%, and 44.94%, respectively. However, in the reference population, completeness of the pedigree is more comprehensive up to fourth generation (66.06%). In our study, mean equivalent complete generation was found to be 2.04 and 4.32 for the whole and reference populations, respectively (Figure 1). However, higher estimate than the present study was reported in Malpura sheep [20], whereas, lower estimates than the present study were reported in Moghani and Segurena sheep [3,4]. The mean equivalent complete generation had substantial impact in obtaining the accurate estimates of inbreeding and also found to be vital in the precise estimation of genealogical parameters. The estimate obtained in the present study indicates the moderate depth of pedigree, satisfying level of genetic variability and the evolution over the period. Low estimate of mean equivalent generations may result due to incomplete knowledge of AR and such problems will be encountered during initial phase of any breeding and conservation program.

**Figure 2:**
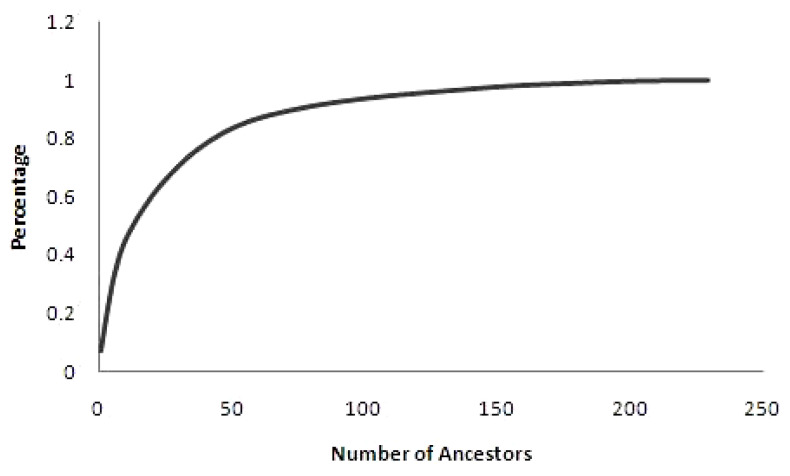
Percentage of genetic variability in the population according to the number of ancestors. It is suggested that a relatively small number of founders explained 50% of the genetic variability in the population.

Almost 50% of the total genetic variability was elucidated by 14 most influential ancestors (Figure 2) with highest individual contribution of 5.96% (ID M178). Results signifies the disproportionate use of particular ancestors for breeding and this might be the major consequence for considerable variation in various traits under genetic improvement program, which aids in breeding of animals by selection method. Similar values were obtained in Malpura (13) and Bharat Merino (14) sheep [21,22]. However, higher values than our study were noted in Iran-Black (46), Segurena (425), Santa Ines (69), and Kermani (33) breeds of sheep [3,4,22,23].

In the reference population, number of founders was 232, and the effective numbers of founders was found to be 47 which represent 20.25% of founders (Table 2). The effective number of founders had contributed significant share for the reference founders. It implies the existence of vast gene pool in the reference population. Information on effective founder is a relevant tool in identifying and managing the inbreeding levels in the flock. Various studies reported different number of effective founders in various sheep breeds viz., 81.1 in Xalda sheep, 143 in Moghani sheep, 1,120 in Segurena sheep, 58 in Malpura sheep, 55 in Bharat Merino, 20 in Valachian sheep, 86 in Zandi and 40 in Afshari sheep [2–4,20,21,24–26].

In the present study, effective number of ancestors was observed to be 37. It was suggested that this parameter enriches the knowledge conferred by the effective number of founders in which it provides the information of loss of genetic variability through unbalanced use of breeding animals and it was also opined that ratio of effective number of founders to the effective number of ancestors is useful in assessing the erosion of genetic diversity because of bottle necks among the base and reference populations and the severity of bottleneck is proportional to this ratio [7]. In the present study, we obtained a f_e_/f_a_ ratio of 1.27. The marginal contribution of all ancestors should be unity, and the f_a_ value should always be lower or equal to the f_e_ [22]. Similar results were noted in various sheep breeds [3,4,21]. Differences in the results may be attributed to the differences in the population structure of the flocks, pedigree depth and completeness, breeding policies implemented and extreme use of particular animals for breeding.

It is presumed that effective population size is considered as number of animals that breed in an ideal population and engender the equal amount of inbreeding in the population under study [27]. The realized effective population size (Ne_r_) noted in the study as 91.56±1.58 (Table 3). Similar results were reported in Malpura and Bharat Merino breeds of sheep [20, 21]. Similarities in the results may be ascribed to the comparable breeding practices adopted in the improvement and conservation of this breed. Lower estimates than the present study were reported in Zandi and Afsari breeds of sheep as 71 and 50, respectively [25,26]. To maintain genetic diversity and to prevent the erosion of genetic variability by genetic drift a number of 500 animals are necessary [1]. Later FAO prescribed a size of 50 as a critical number, however, we maintain 400 breeding animals to manage the genetic diversity and the number is inconsistent as it varies with time and amount of inbreeding in the flock over the time [28].

The population under study had experienced the inbreeding coefficient (*F*_i_) in the reference cohort as 1.38%. For the complete pedigree, percent of inbred animals in second generation was found to be 3.59% and it rose to 79.77% at ninth generation (Table 4). Similarly, percent inbreeding in the pedigree is 0.61% at second generation and increased to 1.64 at third generation and declined with the number of generations (Table 4).

Similar estimate of inbreeding coefficient (*F_i_*) % was reported by in various sheep breeds [25,26]. Whereas, higher value was reported in Malpura sheep (3.32%) [20]. In the reference cohort the 72.04% of animals had inbreeding coefficient in the range of 0 to 6.25%, whereas, 27.95% of animals had zero inbreeding coefficients. Low levels of inbreeding in the population under study is noticed, efficient mating strategies includes accurate preparation of sire lines and evading the breeding of animals with similar sire lines had aided in managing the inbreeding in the population. Instead of implementing best mating plans, 2.51% of matings were between half sibs (142) and 0.42% matings were between parent-offspring (24) in the whole pedigree. This is also proved by increased inbreeding coefficient over the years (Figure 1a).

The AR among the animals for reference population pedigree was found to be 2.48%. The AR values shown tendency to increase over the years (Figure 1a), and the results also suggested that the AR value increased from 1% to 2% (Table 4) at fifth generation. AR is another vital parameter in genetic diversity analysis like inbreeding coefficient. It provides the information on role of each individual in contributing to the genetic diversity to the population, genetic diversity is proportional to the estimate of AR value, and higher AR value implies higher contribution of individuals to the genetic diversity. The mating strategies should be prepared with utmost care when higher AR values observed in the flock; otherwise, breeding of animals with higher AR values may result in animals with high AR value at objectionable level [2].

In the present study, it is found that increase in inbreeding by maximum generation was 0.19%, and by complete generation was 0.74%. It is suggested that the pedigrees with insufficient information may result in inaccurate estimation of genealogical parameters such as inbreeding coefficient and AR (Figure 1b) and hence, utmost care should be taken in managing the pedigree records in the database which will help in accurate estimation of inbreeding levels in the flock.

Individuals born during 2009 and 2012 were considered for the estimation of GI and the average generation length obtained in the present study was 3.38±0.10 years. Ram to daughter pathway was lowest (2.58 years) and highest for ewe to son (4.11 years) (Table 5). However, the estimates of generation length reported in earlier studies were ranged from 2.58 to 4.98 in various breeds of sheep.

Decrease in the GI may result in better economic returns because of improved annual genetic gain and this outcome is the choicest one for the production enterprises. However, shortened stayability of individuals in the flock especially rams will intensify the genetic variability losses as the genetic contribution of those animals will be less. Hence, animal conservation programs should be balanced and the breeding strategies should be planned to achieve lowered GIs with decent annual genetic gains along with sustained genetic variability in the flock.

**Figure 3:**
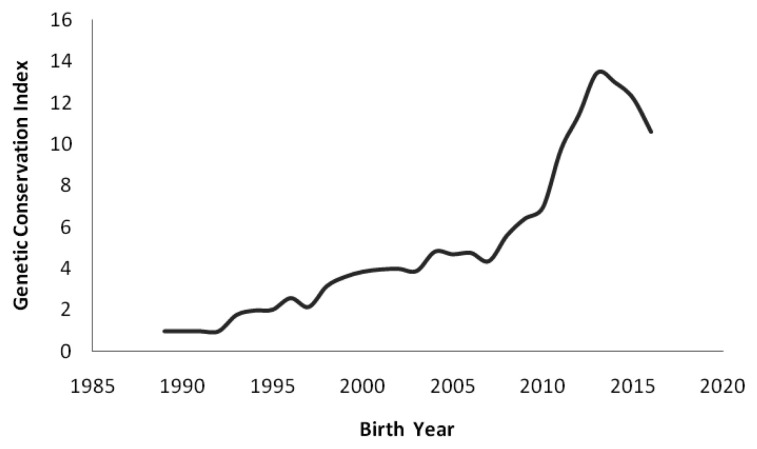
Evolution of the mean genetic conservation index (GCI) of animals by birth year. The index is computed from the genetic contributions of all the identified founders. The mean GCI values increased especially during the year 2012–13 and then decreased thereafter, the possible reason for this inconsistence is due to addition of few unrelated animals in nucleus breeding.

Average GCI of animals by birth year are presented in Figure 3. The index is helpful in describing the individuals as parents which intensifies the presence of founder genes in the next generations. Generally, an ideal individual receives equal contribution from all the ancestors of base population and the individual with higher GCI values, the higher the values of an animal for conservation. However, this index has a disadvantage as it did not consider for pooling of any breeding to non-founder animals in following generations in a pedigree [6].

The mean GCI values increased especially during the year 2012–13 and then decreased thereafter, the possible reason for this inconsistence is due to addition of few unrelated animals in nucleus breeding flock during the year 2013–14, which lowered the mean GCI values of individuals in the following years.

Least-squares means for the traits under study were in agreement with the findings of earlier reports [29] and the estimates for BWT, WW, and 6MW were as follows: 3.06± 0.05, 12.35±0.37, and 17.44±0.24 kg, respectively. Two animal models with either use of *F_i_* or Δ*F_i_* were utilized in the study to know the impact of inbreeding on growth traits of Nellore sheep. It is observed from the analyses that year of birth and sex of lamb was major sources of variation in the studied traits. Ewe weight at lambing had a significant influence on weights at birth and three months. Either *F_i_* or Δ*F_i_* had no influence on traits and there are no observable changes in the fit of model when *F_i_* and Δ*F_i_* used as a covariate and the p-values observed in analyses for BWT, WW, and 6MW as 0.22, 0.51 and 0.24, respectively (Table 6). Inclusion and exclusion of *F_i_* or Δ*F_i_* had no effect on the estimates of variance components and genetic parameters in our study (Table 6). However, earlier researchers observed significant impact of inbreeding on growth traits in various breeds of sheep [21,22].

## IMPLICATIONS

In the current investigation, pedigree analysis is used to monitor the extent of genetic variability in the closed nucleus flock of Nellore sheep and the parameters obtained in the present study were reasonably good, but estimates obtained from the probability of gene origin suggested the erosion of part of genetic variability in the reference population as compared to founder population. Inbreeding coefficient and AR for the reference population were relatively lower in magnitude. Besides, non-significant influence of inbreeding on growth traits, majority of individuals (72.4%) in the reference cohort had inbreeding coefficient values between zero and 6.25% which is a matter of concern. It is imperative to introduce new germplasm in to the flock to minimize the anticipation of detrimental effects of inbreeding on the animals; also it is suggested to design better mating strategies based on the obtained results to prevent the crossing between related individuals which reduce the increased frequency of undesirable effects of inbreeding in the population.

## Figures and Tables

**Table 1:** Animals in the data file and completeness of the pedigree

Items	
Total number of animals	5,663
Proportion of animals with known pedigree	5,031 (88.83%)
Reference cohort (2012–2016)	1,322
Mean equivalent generations	4.23

**Table 2:** Criteria calculated from the probabilities of gene origin for reference cohort

Items	
Total number of founders (f)	232
Effective number of founders (f_e_)	47
Effective number of ancestors (f_a_)	37
Contribution of the main ancestor (%)	0.96
f/f_e_	4.94
f_e_/f_a_	1.27
Founder genome equivalents (f_g_)	22.48
Number of ancestors explaining 50%	14

**Table 3:** Inbreeding, average relatedness and effective population size in the reference population

Items	
Coefficient of inbreeding (*F*_i_) in the reference population (%)	1.38
Proportion of animals with *F*_i_ = 0%	27.95
Proportion of animals with *F*_i_ = 0% to ≤6.25%	72.04
Proportion of animals with *F*_i_ = >6.25% to ≤12.50%	-
Proportion of animals with *F*_i_ = >12.5%	-
Average relatedness (AR %)	2.48
Realized effective population size (Ne_r_)	91.56±1.58

**Table 4:** Mean value of inbreeding (F) and percentage of endogamic animals of Nellore sheep using maximum number of generations traced

Generation	Animals (N)	F (%)	% Inbred	Average F for inbred	Average relatedness (AR)
0	621	0.00	0.00	-	0.16
1	732	0.00	0.00	-	0.75
2	613	0.61	3.59	17.05	1.32
3	569	1.64	14.59	11.26	1.54
4	613	0.84	15.66	5.34	1.85
5	525	1.19	25.52	4.67	2.28
6	451	1.62	48.56	3.34	2.63
7	632	1.54	72.15	2.14	2.63
8	644	1.40	78.88	1.78	2.53
9	262	1.57	79.77	1.97	2.58

**Table 5:** Generation intervals (in years) for the four pathways of the Nellore sheep (cohort born from 2012 to 2016)

Pathway	N	GI±SE (yr)
Ram-Son	21	2.58±0.16
Ram-Daughter	195	2.83±0.54
Ewe-Son	21	4.11±0.45
Ewe-Daughter	195	3.94±0.33
Total	432	3.38±0.10

GI, generation intervals; SE, standard error.

**Table 6:** Least-squares means and the effects of inbreeding on growth traits in Nellore sheep

Trait	V_a_	h^2^	Mean±SE	Effect of *F*_i_	p-value
Birth weight	0.03	0.17±0.02	3.06±0.05	NS	0.22
Three month weight	1.65	0.28±0.02	12.35±0.37	NS	0.51
Six month weight	1.51	0.27±0.02	17.44±0.24	NS	0.24

NS, non-significant; SE, standard error.
